# A very French connection, a brief discourse in commemoration of life and services of the late Louis Pierre Joseph Delpy on his 120^th^ birthday anniversary

**DOI:** 10.30466/vrf.2019.111960.2661

**Published:** 2019-12-15

**Authors:** Keyvan Tadayon, Ehsan Mostafavi, Afshin Hajizadeh, Rainak Ghaderi, Parham Parham

**Affiliations:** 1 *Agricultural Research, Education and Extension Organization (AREEO), Razi Vaccine and Serum Research Institute (RVSRI), Karaj, Iran;*; 2 *Department of Epidemiology, Pasteur Institute of Iran, Tehran, Iran;*; 3 *Department of Internal Medicine, School of Medicine, Qazvin University of Medical Sciences, Qazvin, Iran*

**Keywords:** Archives of Razi Institute, Iran, Louis Pierre Joseph Delpy, Vaccine, World Health Organization

## Abstract

Razi Vaccine and Serum Research Institute (RVSRI) turned 95 years old in 2015. Majority of the animal infectious diseases such as rinderpest and anthrax that used to frequently strike the historic Persia are now gone for good or under control owing to the pioneering researches conducted at the institute in the early-mid 20^th^ century in the field of vaccine manufacturing. The earliest such scientific contributions, were truly made by the French eminent veterinarian Dr. Louis Pierre Joseph Delpy who joined the institute in 1931. In his 18 year-long directorship tenure he taught his colleagues fundamentals of vaccinology, basics of modern epidemiology, essentials of infectious disease control disciplines, the art of scientific writing and much more things that changed the institute for ever. This paper reviews the events and turning points in the first 25 years of service of the institute in a chronological way and remarks Delpy’s principle involvements in all of these on the occasion of the 120 anniversary of his birth. At the entrance of the institute headquarter building where his bronze bust is placed, visitors can see a memorial etched plate that reads “*... The architect of Razi and founder of Archives De L’Institute Razi (Archives of Razi Institute) was an enthusiastic scientist with a creative mind. … For the Razi community, Dr Delpy is gone but not forgotten.”*

## Preludes to 1930

In 1916 rinderpest extensively struck Iran, a disaster that was followed by more epidemics of the disease coming back again in spring 1924 reported first in Tafresh, Arak and Qom and central Iran. This time, extent of the affected territorial land was unprecedentedly extensive fearing total purge of national cattle stocks.^1^ The parliament was flooded with farmers appealing complaints and petitions sent from all over the country. Nevertheless, the Iranian government was also urged by the newly-found Committee of Hygiene of the League of Nations (the precursor of the today World Health Organization) and the Office International des Epizooties (OIE) to take urgent practical actions in establishment of appropriate infrastructure required for control of animal infectious diseases. These events paved the way for foundation of a new governmental body, the then "Hessarak serum making and veterinary studies institute".^2^ In fact, the roots of the today's Razi Vaccine & Serum Research Institute (RVSRI) otherwise known as "Razi institute", “Hessarak serum making institute” and “Hessarak institute” took hold in the old Persian army post office and remount service beside the small village of Hessarak on the premises of Kamalabad, a suburb of Karaj. The big move came on when sometime around 12 o’clock on 6^th^ February 1926, the parliament passed the "Animal pesticides and serum making institute" Act.^3^^,^^4^ This was a historic success for Abdol Hossein Teimourtash and Mostafa Gholi Bayaat, Secretary of Agriculture, Commerce and Public interests and his deputy in the government cabinet of the time. Razi was actually the first national center founded to conduct research specifically into animal infectious diseases and, therefore, counts among the oldest Asian research institutes still active in this field.^1^

**Fig. 1:**
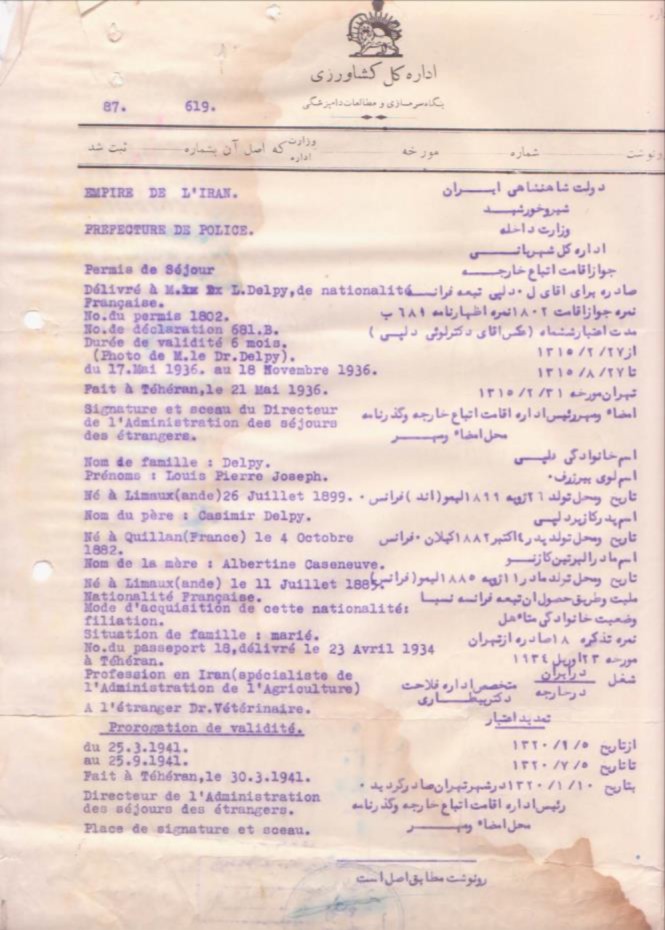
Residence permit issued by the Iranian Home Office in favor of L.P. Delpy and his family dated 1936 (obtained from Razi institute office of personnel records)

Between January 1926 and June 1929 the new-born institute experienced an instability period in its activities due to lack of a clear mandate worsened by lack of a centralized steering leadership. A major breakthrough came in the mid-1929 when a second memorable act passed by the parliament let the government to employ a European veterinarian to direct the institute. The act was clear in employing a French veterinary practitioner expert in bacteriology and vaccine and serum manufacturing. In September 1930, therefore, Mahmoud Fateh the commissioner of the National Economy Department, who was himself a graduate of the French National School of Agriculture Superior Grignon, was assigned to Europe to buy a "sugar refinery plant" to be the first of such in Iran.^3^ As instructed in Tehran, Fateh had a second mission so while staying in Paris, he initiated serious efforts to enroll four French experts in veterinary bacteriology, forestry, botany and horticulture. Fateh made the arrangements for public advertising the job offers with hopes to receive positive response from Toulouse Veterinary Faculty graduates. The surprise came up when a young well-trained Toulouse graduate turned up in Paris coincidently with a plan to finish up with writing his doctoral dissertation. This was Louis Delpy who came from French stock. He was born in Limoux (Aude), eastern France on July 26, 1899 to a very young family with his father, Casimir Delpy, 17 and his mother, Albertine Caseneuve, only 14 at the time of their son's birth (Fig. 1). 

We do not know that much about his childhood. He studied at the Veterinary Faculty of Toulouse and qualified in 1922. The Parisian parasitology research laboratory under Professor Brumpt and the Alfort-based National Veterinary Research Laboratory lead by Professor Vallee where other stops Louis spent two more years of internship and research activities at them. He then approached the French ministry of oversees (Ministère de l'Outre-Mer) to work as a veterinary researcher and was dispatched to the French West Africa. In Bamako he was given opportunity to work closely on local diseases of trypanosomiasis, anthrax and rinderpest. Delpy was lucky enough to work aside with the late G. Curasson, a world-known expert on rinderpest. He played a major role in development of the formaldehyde spleen vaccine greatly successful against rinderpest, an innovated type of vaccine that was known as Curasson-Delpy.^5^

A number of young Iranian students with scholarships awarded by the government were studying at the Veterinary Faculty of Toulouse while negotiations between Fateh and Delpy continued in Paris. This fortunate coincidence positively influenced the ongoing talks as Louis himself was a Toulouse graduate.^2^ At the end, it was Delpy’s destiny to accept the offer, something that changed his whole life for the next twenty years. Very soon, Delpy cruised a long way east from France to Persia that in its final stage passed through Russia. He landed at Pahlavi (now Bandar Anzaly) port at the Caspian Sea, northern Iran. He arrived eventually at Tehran in March 1931 and warmly welcomed by the Iranian Minister of Agriculture, now Mostafa Gholi Bayaat, who was another Iranian graduate of the Ecole Nationale Supérieure d'Agriculture de Grignon. Delpy was briefed by the reformist agriculture officer on his clear intension to establish an institute for research in diagnosis of animal diseases and manufacturing vaccines against them.

## Delpy’s achievements

Delpy was left by Mostafa Gholi Bayaat to decide whether to give up the Hessarak premises and move to the Karaj Agriculture school fields or stay on the institute original lands in Hessarak for renovation and construction works. Delpy opted to stick to Hessarak. Very soon his construction plans to build up a microbiology laboratory as well as further facilities for making serum and vaccine were submitted to the ministry which were all accepted and funded with almost no request for any change. A year later the first new building of the institute was built and named “Diagnostic Laboratory of Animal Diseases”.

With arrival of the required equipment, chemicals and books from Europe ordered in advance by Delpy, things were all in place to get the facility operational. Despite logistical difficulties in supply of equipment and chemicals due to WWII, in the following years more laboratories were constructed and came to service. With joining of new Iranian graduates coming from abroad or the veterinary school in Tehran, Delpy’s technical working team was extended as well.

In April 1932, Delpy was appointed the general director of the newly-found state veterinary organization (formerly known as veterinary service), a position he hold until 1935.^6^

In April 1937, Delpy developed a medical condition in his right arm that brought him discomfort and pain, something that was acquired as a result of his laboratory and clinical experiments with anthrax agent.

In February 1939 he was assigned to France to conduct the health checks on pure-breed cattle destined for animal breeding in Persia. While in Paris, Delpy decided to seek medical treatment for his still unhealthy right arm. On top of these, in September 1939, while still on sick-leave, Delpy was asked to report to the French Defense ministry for a three-month long term of duty. His leave of absence was therefore extended by officials until January 1940 when he finally returned to Iran.

Beside his main commitment as general director of the institute, his work contract had tasked him to participate in tutorials at the newly-founded agriculture, medicine and veterinary colleges of Tehran university a responsibility he committed seriously until the very last years of his stay in Iran.^6^ This was at the cost of his regular weekly visits to Tehran and commuting some 90 kilometers every time between the institute and the college through the unsafe single road of the time, long before the today’s Karaj, Hemmat and Hakkim highways are built.

As a further responsibility, Delpy was often asked to visit cities and villages all over the country to address the reported epidemics of animal diseases, in many cases unknown to his Iranian colleagues. These field visits and experiences improved Delpy’s already great deal of epidemiology knowledge on infectious diseases of animals in Persia, something that was later mirrored in his brilliant book published in 1938 titled “Infectious diseases of farm animals in Persia”.^7^ A very informative, if not the best, handbook ever written in the modern history of veterinary medicine in this country. In his book and for each disease, Delpy provided detailed reports of his observations, success or failure in curing of the clinical cases and the actions taken by him to control the disease dissemination in the field.

In 1939, Delpy founded “Archives de L’Institut Razi” (Archives of Razi Institute), the first recorded ever published scientific periodical in the country. Within the ten volumes of the journal that were reproduced under his editorship, Delpy contributed in more than 40 articles as the principle or main co-author. This included nine articles on bacterial and viral diseases of animals and human, eight on ticks and eight on protozoa. These were all reflections of the researches conducted at the institute by himself and his Persian colleagues.^5^ In reality, as quoted by Kaweh, Delpy was a generous and gentleman scientist, a one who helped his unexperienced teammates having no knowledge of scientific writing, with manuscripts of his own getting published under their names. This appeared to be a successful policy to mature the journal he founded himself.^5^

In 1939 and under the auspices of the institute, Delpy visited Cairo, Egypt to help Egyptians with technology transfer of rinderpest vaccine production.

In 1946 Delpy in a commentary published on the occasion of the 16^th^ anniversary of the institute, praised establishment of Hessarak institute as an appropriate measure taken by the government in combat of the diseases threatening the national livestock resources.^8^ He reiterated, in a country where civilian veterinary practitioners were not available and one would not expect a consent from local community of shepherds, herdsmen and farmers, then application of modern approaches in control of animal diseases would be absolutely impractical. It was, therefore, not plausible to employ measures such as sanitary police system (e.g. compulsory declaration of diseased animals, quarantine, disinfection, culling etc.) in Persia. Nevertheless, the traditional nomadic breeding methods that favor disease transmission but had been employed for very many decades, were another challenge. In such environment, focusing on elimination of disease foci through long-term strategies were actually not practical and it was much easier to provide some level of protection against infectious disease in the livestock at large through mass vaccination schemes.^8^ This was more adoptable with the country’s political and administrational atmosphere at the time. Delpy continued that from 1931, when he arrived Iran, till 1934 the institute activities were limited to few temporary installations but with required funds received from Tehran the construction plan progressed and by 1940 the infrastructure was almost completed.

**Fig. 2:**
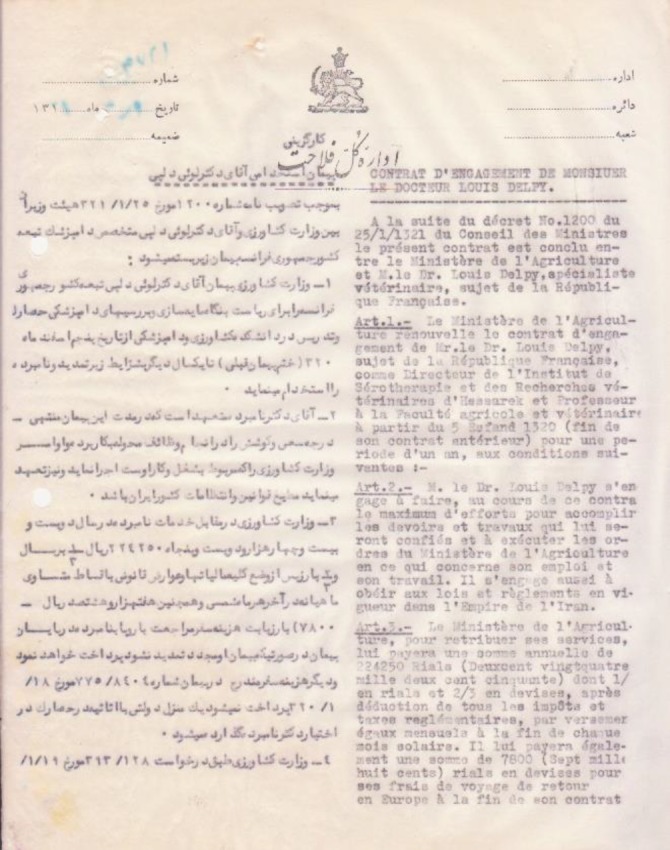
The front page of L.P. Delpy’s employment contract with Iranian Department of Agriculture, dated 1939 (obtained from the Razi institute office of personnel records)

The excellent high quality services Delpy provided during his stay in Persia, convinced the Ministry high commends to apply for extension of his contract. Besides, the flourishing bilateral political ties between the two States were deep enough to let the French Ministère de l'Outre-Mer renew their employee’s assignment. His initial 5 year-long work contract (1931-1936) was therefore extended by the government and approved by the national parliament for several more times (Fig. 2) until 1951 when this came to an end on his own wish.

Parasitology was one of the Delpy’s main personal research interests, a discipline he had a deep knowledge.^9^^-^^12^ He made a big contribution to the field of ruminant ectoparasites (ticks) in Persia.^13^^-^^16^ A fact that is reflected by the published literature.^16^ Delpy played a pivotal role in development of many of the biologicals in the institute’s production portfolio. Vaccines such as rinderpest, anthrax, pasteurellosis, salmonellosis, tetanus and sheep pox are examples of those Delpy contributed in obtaining their production technology.

**Fig. 3:**
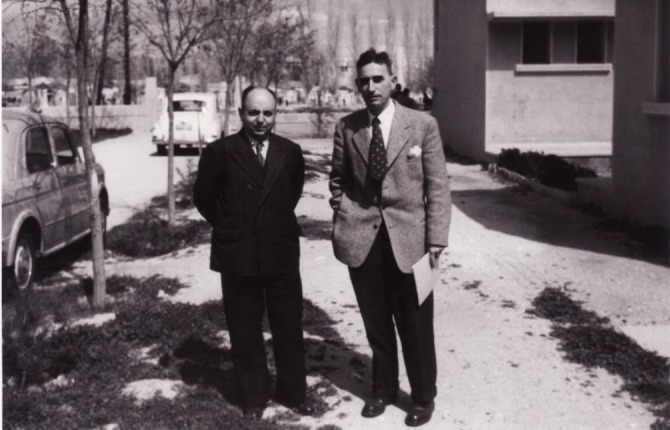
A middle-aged L.P. Delpy poses for a photo during a scientific visit to Morocco. Delpy was accompanied by his Razi colleague and close friend the Late A. Rafie (the man on his right with black suit), (Photograph courtesy of Dr. Rafie, junior)

In October 1949 Aziz Rafiee, Delpy’s deputy took over directorship of the institute but Delpy remained to finish with his work contract until February 1951 before returning to France (Fig. 3).

Once back home, he worked for several years at Mérieux as scientific and technical director until 1955 when he gave up his post to take a full-time retirement and live peacefully in his villa in Cannes, southern France. He, however, remained active conducting occasional consultancy missions for OIE and/or FAO. Delpy visited Iran for few more times (1955, 1957, 1961, 1966 and 1973) since he left Razi on invitations by the agriculture ministry and spent some time in all these visits at the institute to reunite with his former colleagues and exchange views on progress in new researches at the institute.

In October 1973 Delpy gave his last dramatic visit to Razi. In May 1974 at the age of 75 at his personal residence in Cannes, Delpy breathed his last, leaving behind a legacy of honor and service to people of Persia. Delpy was survived by his wife, Valentine, his son, Jacque and his daughter, Claudie. Delpy was a double medalist who was decorated by French government with Légion d'honneur twice.

## Acknowledgements of Delpy’s work impression

**Fig. 4:**
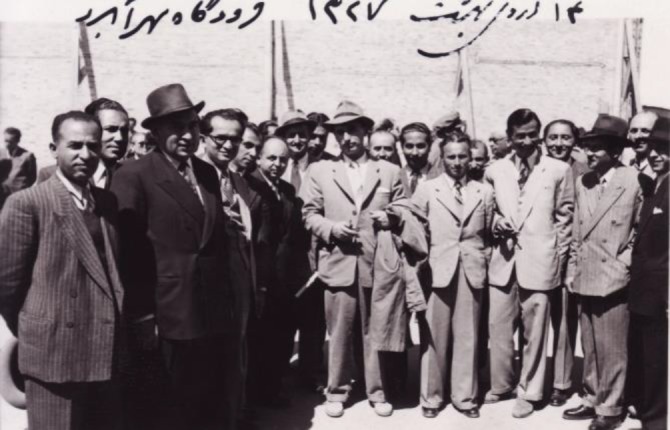
L.P. Delpy (center, holding his raincoat) is greeted by his Razi colleagues. Mehrabad airport, Tehran, May 1948 (photograph courtesy of Dr. Rafie, junior)

In recognition of the services rendered by Delpy, the first laboratory of the institute was named after him. In a landmark obituary published in 1974 specifically to mark Delpy’s decease, Kaweh, Delpy’s assist and close friend at Razi, wrote about his *idee fixe* which was: “life is short and our duties are just too many so try focus on what you are doing now rather than concerning about what needs to be done next”. Those privileged men who worked in his unit or lucky scientist who spent only some time chatting with Delpy, would always remember him as a very exceptionally charismatic mentor (Fig. 4).

**Fig. 5:**
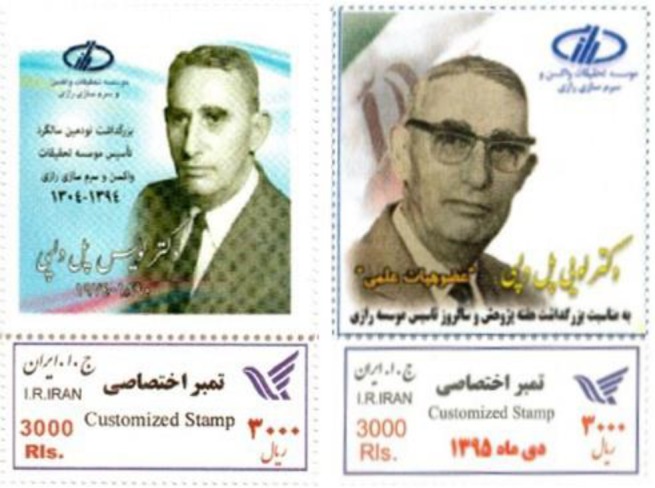
Commemorative postage stamps, issued by I.R.Iran in 2015 and 2016, featuring L.P. Delpy, 1899-1974

In 2015 and 2016, two single commemorative postage stamps were commissioned to honor Delpy (Fig. 5). In January 2015 and at the 90^th^ anniversary ceremony of the institute, the bronze busts of Delpy and his three close aids and major figures in history of Razi, Aziz Rafiee, Morteza Kaweh and Hossein Mirshamsi, were happily inaugurated.

## Conclusion

Delpy remains an inspirational picture of a genuine scientist who gave up the convenience and comfort of a Parisian wealthy life to make a big difference for a nation he respected a lot and his circle of colleagues who would tremendously miss him.
